# Effects of Vitamin D Supplementation on Bone Turnover Markers: A Randomized Controlled Trial

**DOI:** 10.3390/nu9050432

**Published:** 2017-04-27

**Authors:** Verena Schwetz, Christian Trummer, Marlene Pandis, Martin R. Grübler, Nicolas Verheyen, Martin Gaksch, Armin Zittermann, Winfried März, Felix Aberer, Angelika Lang, Gerlies Treiber, Claudia Friedl, Barbara Obermayer-Pietsch, Thomas R. Pieber, Andreas Tomaschitz, Stefan Pilz

**Affiliations:** 1 Department of Internal Medicine, Division of Endocrinology and Diabetology, Medical University of Graz, Graz 8036, Austria; verena.schwetz@medunigraz.at (V.S.); marlene.pandis@medunigraz.at (M.P.); martin.gruebler@gmx.net (M.R.G.); martin.gaksch@gmail.com (M.G.); felix.aberer@medunigraz.at (F.A.); angelika.lang@stud.medunigraz.at (A.L.); gerlies.treiber@medunigraz.at (G.T.); barbara.obermayer@medunigraz.at (B.O.-P.); thomas.pieber@medunigraz.at (T.R.P.); stefan.pilz@medunigraz.at (S.P.); 2 Swiss Cardiovascular Center Bern, Department of Cardiology, Bern University Hospital, University of Bern, Bern 3011, Switzerland; 3 Department of Internal Medicine, Division of Cardiology, Medical University of Graz, Graz 8036, Austria; nicolas.verheyen@medunigraz.at (N.V.); andreas.tomaschitz@gmx.at (A.T.); 4 Clinic for Thoracic and Cardiovascular Surgery, Heart and Diabetes Center NRW, Bad Oeynhausen 32545, Germany; azittermann@hdz-nrw.de; 5 Clinical Institute of Medical and Chemical Laboratory Diagnostics, Medical University of Graz, Graz 8036, Austria; Winfried.Maerz@synlab.com; 6 Department of Internal Medicine, Division of Nephrology, Medical University of Graz, Graz 8036, Austria; Claudia.Friedl2@klinikum-graz.at; 7 Bad Gleichenberg Clinic, Bad Gleichenberg 8344, Austria

**Keywords:** vitamin D supplementation, bone turnover markers, osteocalcin, procollagen type 1 N-terminal propeptide, crosslaps, bone-specific alkaline phosphatase

## Abstract

Bone turnover markers (BTMs) are used to evaluate bone health together with bone mineral density and fracture assessment. Vitamin D supplementation is widely used to prevent and treat musculoskeletal diseases but existing data on vitamin D effects on markers of bone resorption and formation are inconsistent. We therefore examined the effects of vitamin D supplementation on bone-specific alkaline phosphatase (bALP), osteocalcin (OC), C-terminal telopeptide (CTX), and procollagen type 1 N-terminal propeptide (P1NP). This is a post-hoc analysis of the Styrian Vitamin D Hypertension Trial, a single-center, double-blind, randomized, placebo-controlled trial (RCT) performed at the Medical University of Graz, Austria (2011–2014). Two hundred individuals with arterial hypertension and 25-hydroxyvitamin D (25[OH]D) levels <75 nmol/L were randomized to 2800 IU of vitamin D daily or placebo for eight weeks. One hundred ninety-seven participants (60.2 ± 11.1 years; 47% women) were included in this analysis. Vitamin D had no significant effect on bALP (mean treatment effect (MTE) 0.013, 95% CI −0.029 to 0.056 µg/L; *p* = 0.533), CTX (MTE 0.024, 95% CI −0.163 to 0.210 ng/mL, *p* = 0.802), OC (MTE 0.020, 95% CI −0.062 to 0.103 ng/mL, *p* = 0.626), or P1NP (MTE −0.021, 95% CI −0.099 to 0.057 ng/mL, *p* = 0.597). Analyzing patients with 25(OH)D levels <50 nmol/L separately (*n* = 74) left results largely unchanged. In hypertensive patients with low 25(OH)D levels, we observed no significant effect of vitamin D supplementation for eight weeks on BTMs.

## 1. Introduction

Bone remodeling is regulated by a variety of systemic and local factors as well as nutritional factors including calcium and vitamin D [1]. As such, vitamin D deficiency is considered to be detrimental to muskuloskeletal health. It is historically known that vitamin D deficiency contributes to disturbed calcium metabolism with consequently increased parathyroid hormone (PTH) levels that may ultimately lead to rickets [2] and osteomalacia. While vitamin D supplementation is currently widely used for the purpose of preventing and treating musculoskeletal diseases, it has to be acknowledged that data on vitamin D, bone mineral density (BMD), and fracture risk are abundant but heterogeneous. 

In a meta-analysis of 23 studies, a small benefit of vitamin D supplementation on BMD at the femoral neck with heterogeneity among trials could be observed, but no effect at any other site was reported [3]. This meta-analysis leads to the conclusion that continuing widespread use of vitamin D for osteoporosis prevention in community-dwelling adults without specific risk factors for vitamin D deficiency might not be appropriate [3].

In light of the contradictory evidence published on vitamin D supplementation and BMD and fracture risk, more data on faster changing parameters with a more rapid reaction time than BMD and fracture development are needed to detect more subtle effects of vitamin D on bone. At the time when structural bone markers such as low BMD, rickets, or osteoporosis are diagnosed, bone health may already be irreversibly damaged, providing the rationale to investigate changes in bone turnover markers (BTMs) upon vitamin D supplementation. There are, however, relatively limited and inconsistent data available regarding vitamin D effects on BTMs [4]. 

To summarize the published data on this topic, the majority of vitamin D studies did not evaluate BTM in their assessment of vitamin D deficiency and bone health, a knowledge gap addressed by the European Food Safety Authority (EFSA) in their scientific opinion paper published in June 2016. The EFSA panel considered that “more research is needed to establish the relationship between responses of bone markers (e.g., osteocalcin, bone ALP, and urine N-telopeptide crosslinks) to changes in vitamin D status” [4]. 

Bone turnover markers (BTMs) can be divided into two groups [5]: markers of bone resorption—C-terminal telopeptide (CTX)—and of bone formation—i.e., osteocalcin (OC), bone-specific alkaline phosphatase (bALP), and procollagen type 1 N-terminal propeptide (P1NP). In the context of vitamin D deficiency, bALP especially is considered of utmost relevance, as its elevation is, together with the appearance of skeletal and radiologic changes, a criterion for the diagnosis of rickets [6,7] and osteomalacia [8]. 

Following the above-mentioned recommendation of the EFSA panel, we performed a post-hoc analysis of the Styrian Hypertension Study, a vitamin D randomized controlled trial (RCT) including 197 hypertensive patients with low levels of 25(OH)D (indicator of vitamin D status), to elucidate the effect of vitamin D supplementation on BTMs. For this purpose, the effects of vitamin D supplementation versus placebo on bALP, OC, P1NP, and CTX were investigated as primary outcome measures. Additionally, cross-sectional analyses were carried out, investigating whether baseline levels of bALP, OC, P1NP, and CTX are correlated with 25(OH)D and 1,25(OH)2D3 (the active, hormonal form of vitamin D). We also performed subgroup analyses in patients with 25(OH)D levels <50 nmol/L (divide by 2.496 to convert nmol/L to ng/mL) as well as sex-stratified analyses. We consider this RCT suitable for evaluating vitamin D effects on bone markers as we could previously already document that vitamin D supplementation significantly increased 25(OH)D and decreased PTH levels in this patient cohort.

## 2. Materials and Methods 

### 2.1. Study Design

This study is a post-hoc analysis of the Styrian Vitamin D Hypertension Trial, a single-center, double-blind, placebo-controlled, parallel-group study performed at the Medical University of Graz, Graz, Austria [9] and registered at EU Clinical Trials Register (http://www.clinicaltrialsregister.eu, accessed 16 February 2011, EudraCT number 2009-018125-70) and at clinicaltrials.gov (ClinicalTrials.gov Identifier NCT02136771). The design and methods have been published previously [9]. This trial’s publications adhere to the Consolidated Standards of Reporting Trials (CONSORT) 2010 statement [10]. 

### 2.2. Study Participants

Individuals aged 18 years or older with diagnosed arterial hypertension and a serum concentration of 25(OH)D below 75 nmol/L were eligible for study participation. Arterial hypertension was defined according to current guidelines [11] or in patients on antihypertensive medication. As previously published [9,12,13], exclusion criteria included hypercalcemia, pregnant or lactating women, acute coronary syndrome, cerebrovascular events within the previous two weeks, drug intake as part of another clinical study, an estimated glomerular filtration rate <15 mL/min per 1.73 m^2^, a change in antihypertensive treatment during the previous four weeks or planned change of antihypertensive treatment, diseases with an estimated life expectancy of less than one year, 24-h systolic blood pressure >160 mmHg or <120 mmHg, 24-h diastolic blood pressure >100 mmHg, any clinically significant acute disease requiring drug treatment, chemotherapy or radiation therapy, or a regular intake of >880 IU of vitamin D daily during the last four weeks in addition to the study medication. All study participants gave written informed consent prior to study inclusion. The study was approved by the ethics committee of the Medical University of Graz, Austria, and was designed to comply with the Declaration of Helsinki.

### 2.3. Intervention

The study medication was filled into numbered bottles following a computer-generated randomization list. Randomization was carried out using a web-based software called Randomizer powered by the Institute for Medical Informatics, Statistics and Documentation, Medical University of Graz, Graz, Austria, with good clinical practice compliance as confirmed by the Austrian Agency for Health and Food Safety. Eligible participants were randomly assigned in a 1:1 ratio to receive either 2800 IU of vitamin D3 (Oleovit D3, Fresenius Kabi Austria, Austria), or a matching placebo administered both orally by seven oily drops per day for eight weeks. Permuted block randomization with a block size of 10 and stratification according to sex was performed. All investigators/authors enrolling participants, collecting data, and assigning intervention were masked to participant allocation.

### 2.4. Outcome Measure

This is a post hoc analysis of the Styrian Vitamin D Hypertension Trial investigating the between-group differences in BTMs at study end with adjustment for baseline values [14].

### 2.5. Measurements

Patient interviews, physical examinations, and blood sampling were performed at study visits between 7 a.m. and 11 a.m. after an overnight fast. Ambulatory blood pressure measurements and 24-h urine collections were started the same day lasting until the following day, when eligible study participants were randomized and intake of study medication was initiated. Serum levels of 25(OH)D were measured by a chemiluminescence assay (IDS-iSYS 25-hydroxyvitamin assay; Immunodiagnostic Systems Ltd., Boldon, UK) with an intraassay and interassay coefficient of variation (CV) of 6.2% and 11.6%, respectively. Total OC (intra- and interassay CV, 0.5% and 1.4%, respectively; analytical range, 0.5–300 ng/mL) was measured by electrochemiluminescence immunoassay (Roche Diagnostics, Mannheim, Germany) according to the manufacturer’s instructions detecting all forms of OC with a similar affinity. CTX (intra- and inter-assay CV, 2.0 and 4.2%, respectively; analytical range, 0.01–6 ng/mL) levels were measured by electrochemiluminescence immunoassay (Cobas, Roche Diagnostics, Mannheim, Germany). bALP (interassay CV: 5.2%) was measured by means of a spectrophotometric immunoassay (IDS-ISYS Ostase BAP; Immunodiagnostic Systems Ltd. [IDS Ltd.], Boldon, Tyne & Wear, UK). P1NP was determined by (interassay CV: 2.7%) means of an automated electrochemiluminescence immunoassay (ECLIA; Roche Diagnostics, Mannheim, Germany). A chemiluminescence immunoassay (IDS-iSYS 1,25VitDXp; Immunodiagnostic Systems Ltd., Boldon, UK) was used to measure 1,25(OH)2D3, with an intra-assay and inter-assay CV of 6.4–12.1% and 6.6–9.6%, respectively. Details on laboratory measurements have been published previously [9]. 

### 2.6. Data Analysis

Continuous data following a normal distribution are depicted as means with standard deviations, parameters with a skewed distribution are shown as medians with interquartile ranges, and categorical data are presented as percentages. Where appropriate, skewed variables were log (e) transformed for parametric analyses. To compare between vitamin D and placebo groups at baseline, the unpaired Student’s *t*-test, the Mann–Whitney U-test, or the chi-square test was used. Differences in BTMs at baseline according to vitamin D levels (i.e., <30, 30–39, <40–49, ≥50 nmol/L) were calculated by analysis of variance (ANOVA). Spearman correlation analysis was performed to evaluate associations between 25(OH)D, 1,25(OH)2D3, PTH, and BTMs.

Analysis of covariance with adjustments for baseline values was applied to test for differences in the outcome variables (i.e., bALP, CTX, OC, and P1NP) between the treatment and the placebo group at the follow-up visit [14]. In addition, we also performed subgroup analyses in patients with 25(OH)D levels <50 nmol/L and conducted sex-specific analyses. Analyses were carried out according to the intention-to-treat principle with no data imputation and inclusion of all participants with baseline and follow-up values of the respective outcome variable. A *p*-value < 0.05 was considered statistically significant. Statistical analyses were performed with SPSS version 23 (SPSS, Chicago, IL, USA). 

## 3. Results

One thousand seven hundred persons were invited to participate in the study, 518 gave written informed consent and were assessed for eligibility, 200 were randomized, and 188 completed the trial. Randomization and follow-up visits were performed between June 2011 and August 2014; a participant flow chart has been published previously [9]. The number of adverse events (i.e., hospitalization or hypercalcemia) did not significantly differ between vitamin D and placebo groups. No study subject died during study participation. In spite of careful monitoring, the exclusion criteria of hypercalcemia were violated in one participant who was randomized with an elevated serum calcium level of 2.69 mmol/L. Due to adherence to the intention-to-treat principle, however, all available participants were included in the final analyses. According to the remaining volume in the bottles, we calculated a compliance of 106 ± 10% in the vitamin D and 106 ± 11% in the placebo group.

In the originally published study, a significant increase in 25(OH)D and a significant decrease in PTH had been observed [9]. In the current study, only participants with at least one available BTM were included (*n* = 197). Available values for bALP, CTX, OC, and P1NP were *n* = 190, 177, 195, and 189 at baseline and 169, 156, 175, and 170 at follow-up, respectively. No data imputation for missing values was performed. The baseline characteristics of the included participants are shown in Table 1. There were no differences at baseline between vitamin D and placebo groups (Table 1) as well as between groups according to vitamin D levels (Table 2).

At baseline, neither 25(OH)D nor 1,25(OH)2D3 correlated with any of the BTMs (Table 3). Analyzing women or men separately did not significantly alter the results (data not shown). OC showed an inverse correlation with serum glucose and HbA1c (Spearman correlations: *r* = −0.267, *p* < 0.001, and *r* = −0.249, *p* < 0.001, respectively). bALP was positively associated with alkaline phosphatase (AP) (Spearman correlation: *r =* 0.737, *p* < 0.001). Correlation analyses between the change in 25(OH)D and the changes in each BTM (delta 25(OH)D, delta bALP, delta OC, delta CTX, and delta P1NP) were performed. However, no significant correlations between delta 25(OH)D and the changes in BTM could be observed (data not shown). 

Vitamin D supplementation did not show a significant effect on bALP, CTX, OC, and P1NP (Table 4). When analyzing patients with 25(OH) levels <50 nmol/L (*n* = 74) or with 25(OH) levels <40 nmol/L (*n* = 45) separately, results remained materially unchanged (data not shown). Analyzing patients with a BMI above the median and below the median, results also remained materially unchanged (data not shown). Analyzing men and women separately, results remained unchanged except for OC in men. OC in men was 11.8 ng/mL (8.8–13.9) (mean and interquartile range) at baseline and 12.1 ng/mL (9.1–15.6) at follow-up resulting in a mean treatment effect of 0.131 (0.020 to 0.242; *p* = 0.022). Analyzing pre- and postmenopausal women separately likewise left results materially unchanged (data not shown). Addressing the issue of multiple testing by dividing the *p*-value for statistical significance by the number of tests, we consider this result as not significant. 

Seventeen patients had a diagnosis of osteoporosis; six patients were on bisphosphonate therapy, one patient was on denosumab therapy while the others did not have specific osteoporosis therapy at inclusion. Sixteen patients were on calcium supplementation. As these treatment regimens were left unchanged throughout the intervention and as exclusion of the respective patients did not alter the results (data not shown), these patients remained in the final analysis. After exclusion of seven patients on antiresorptive bone-active medication, results remained materially unchanged. Again, vitamin D had no significant effect on bALP (MTE 0.013, 95% CI −0.030 to 0.057 µg/L; *p* = 0.540), CTX (MTE 0.021, 95% CI −0.168 to 0.211 µg/L; *p* = 0.823), OC (MTE 0.028, 95% CI −0.055 to 0.111 µg/L; *p* = 0.502), or P1NP (MTE −0.027, 95% CI −0.102 to 0.047 µg/L; *p* = 0.474). 

## 4. Discussion

In this RCT in hypertensive, middle-aged patients without osteoporosis with low 25(OH)D levels, no effect of high-dose vitamin D supplementation on markers of bone turnover—bALP, CTX, OC, and P1NP—was observed. In addition, there was also no significant cross-sectional association between 25(OH)D and BTMs. 

These findings address a knowledge gap on vitamin D and BTMs as stated by the 2016 EFSA report on vitamin D [4]. Previous RCT data on vitamin D supplementation and BTMs were inconsistent, but our data are well in line with previously published studies, who did not show a significant effect of vitamin D treatment on bone turnover as assessed by CTX, OC, bALP, and P1NP [15,16]. The patient cohorts analyzed included healthy young and elderly adults [16] and healthy obese men and women [15]. Likewise, vitamin D supplementation did not have an additional effect on markers of bone metabolism in congestive heart failure patients with low 25(OH) levels who were provided with adequate daily calcium intake [17]. 

The lacking effect on bALP in our RCT is of particular interest because an increased bALP activity is an accepted biomarker in the diagnosis of vitamin D deficiency in the context of rickets [6] and osteomalacia [8]. Patients with vitamin D deficient rickets or osteomalacia show increases in bALP and decreases upon supplementation. It must, however, be acknowledged that vitamin D deficient rickets and osteomalacia is only evident at very low 25(OH)D levels. However, in the present cohort, only four individuals had overt vitamin D deficiency with 25(OH)D concentrations at baseline of below 30 nmol/L; additionally, as opposed to patients with rickets and osteomalacia, the cohort at hand must be considered well-nourished with a sufficient calcium intake. According to the Austrian nutrition report of 2012 [18], Austrian women aged 51–64 had a mean daily calcium intake of 786 mg, Austrian men the same age of 802 mg, lying just below or meeting the estimated average requirements of calcium as recommended by the Institute of Medicine (IOM) [19], respectively. Therefore, the missing effect of vitamin D supplementation on bALP might be attributed to the relatively high 25(OH)D levels and a presumably relatively high calcium intake in our cohort. This hypothesis is also supported by the study by Schleithoff and colleagues, showing no additional beneficial effects of a rise in serum 25(OH)D from approximately 40 to 100 nmol/L in congestive heart failure patients with initially insufficient daily calcium intake receiving an adequate daily calcium supplementation. In their study, the daily intake of 50 mg of vitamin D3 and 500 mg of calcium for 9 months was not superior to 500 mg of calcium alone in reducing BTMs, despite an adequate rise in serum 25(OH)D levels above 75 nmol/L. As our patient cohort at hand is considered to have sufficient calcium intake, a combination of calcium and vitamin D supplementation would likely not have affected our results, although we have to acknowledge that some meta-analyses of RCTs suggest that only a combined vitamin D and calcium supplementation decreases fracture risk, whereas vitamin D supplementation alone may not be effective [4,19]. 

Interestingly, Kuchuk et al. suggested that [20] BTMs might only be affected by vitamin D supplementation at 25(OH)D levels below 40 nmol/L. This hypothesis is based on the fact that a highly significant relationship between serum 25(OH)D and urine deoxypyridinoloine/creatinine (DPD/Cr) was only observed in individuals with 25(OH)D levels below 40 nmol/L but not above this threshold. We addressed this issue by subgroup analyses in individuals with 25(OH)D levels below 50 nmol/L and below 40 nmol/L, but we again found no significant effect of vitamin D supplementation on BTM. Due to a low prevalence of several vitamin D deficient patients we were, however, not able to perform adequately powered subgroup analyses in patients with 25(OH)D levels below 30 nmol/L. 

In line with the lacking effect of vitamin D supplementation on BTMs in the present cohort are the divergent data on the effects of vitamin D supplementation on BMD [21,22,23,24,25,26,27] and fracture risk. In the study by Sanders annual oral administration of high-dose cholecalciferol among older community-dwelling women even resulted in an increased risk of falls and fractures [28]. Evidence from published meta-analyses is mixed. While one meta-analysis is supportive of a beneficial effect of vitamin D on fracture risk reduction [29], two others show no effect of vitamin D alone on diminishing fracture risk, although vitamin D reduced fractures in combination with calcium [30,31]. 

Regarding pathophysiologic effects of vitamin D on bone, it has to be noted that vitamin D receptor (VDR) activation in osteoblasts, osteocytes, chondrocytes, and osteoclasts regulates cell proliferation, maturation, and mineralization as well as bone resorption. Whether the net effect of VDR activation on bone health is beneficial or even harmful, however, remains unclear as mechanistic studies show inconsistent and partially contradictory results [32,33,34,35]. Therefore, despite decades of research on the topic of vitamin D and bone health, there remain several knowledge gaps on this topic with our study supporting the notion that vitamin D supplementation at doses of 2800 IU daily does not have significant effects on bone turnover in patients with low but not severely reduced 25(OH)D levels. 

The limitations of our study include the fact that calcium intake was not determined and the fact that our results are derived from a cohort of patients with hypertension and low 25(OH)D and can thus not be generalized to other study populations for whom the topic might be even more critical, i.e., patients with osteoporosis. The characteristics of our cohort may, however, also be regarded as a strength of the study presented, as vitamin D supplementation in non-osteoporotic patients is often initiated in daily practice and therefore of relevance. Furthermore, BTMs in our study cohort were relatively low, thus indicating rather good bone health. Another limitation is the post hoc analysis requiring caution in terms of data interpretation. Furthermore, the lack of data on BMD and the relatively short study duration are additional limitations to this study. Multiple testing was not a problem in this trial as our findings were not formally significant. Furthermore, clear strengths of our study include its design as an RCT and, as shown previously [9], the confirmation of an effective vitamin D treatment by a significant treatment effect on 25(OH)D and PTH levels and by its relatively large sample size when compared to previous studies on this topic. The validity of our results are emphasized by, in our observed cohort, the previously published well-known associations between markers of bone turnover [36], such as correlations between OC and CTX [37], or P1NP and OC [38]. Additionally, validity is substantiated by confirmation of well-known correlations of AP and bALP [39], OC and HbA1c [40,41], and OC and glucose [42]. 

In conclusion, we did not observe an effect of vitamin D supplementation on bALP, CTX, OC, and P1NP in patients with low 25(OH)D levels and arterial hypertension. Further studies are still needed to evaluate the relationship between vitamin D supplementation and BTM, BMD, and fracture risk in individuals with overt vitamin D deficiency.

## Figures and Tables

**Table 1:** Baseline characteristics of all randomized participants with available bone turnover markers (BTMs) at baseline.

Baseline Characteristics	All (*n* = 197)	Vitamin D (*n* = 98)	Placebo (*n* = 99)	*p*-Value
Age (years)	62.4 (52.9–68.1)	62.4 (54.1–69.2)	61.7 (51.5–67.5)	0.705
Females (%)	47	46	49	0.414
Females postmenopausal (%)	81.5	80.9	82.2	0.540
BMI (kg/m^2^)	29.9 (27.1–33.1)	29.7 (27.5–33.1)	29.9 (26.4–32.9)	0.606
Office systolic BP (mmHg)	142.0 (130.0–152.0)	141.0 (131.5–152)	143.0 (130–152.5)	0.796
Office diastolic BP (mmHg)	86.0 (80.0–94.8)	86.0 (80.0–94.0)	87.0 (78.0–95.0)	0.751
25(OH)D (nmol/L)	54.5 (43.0–64.3)	56.5 (47.5–65.5)	53.0 (38.0–63.8)	0.081
1,25(OH)2D3 (pg/mL)	47.7 (37.0–63.3)	47.9 (37.5–63.3)	46.5 (35.9–63.4)	0.548
PTH (pg/mL)	51.3 (39.6–63.6)	48.9 (39.6–61.9)	51.5 (39.3–65.9)	0.729
bALP (µg/L)	15.9 (13.1–19.9)	15.3 (12.3–18.7)	16.7 (13.3–20.6)	0.087
CTX (ng/mL)	0.18 (0.10–0.29)	0.17 (0.09–0.27)	0.19 (0.11–0.29)	0.401
Osteocalcin (ng/mL)	12.3 (9.1–16.7)	11.7 (9.0–16.6)	13.0 (9.1–18.0)	0.252
P1NP (ng/mL)	37.0 (30.0–50.0)	34.9 (26.2–49.0)	39.7 (31.4–51.0)	0.085
Serum calcium (mmol/L)	2.37 (2.30–2.42)	2.37 (2.3–2.42)	2.38 (2.31–2.42)	0.437
Serum phosphate (mmol/L)	0.94 ± 0.16	0.93 ± 0.17	0.97 ± 0.16	0.089
Diabetes mellitus (%)	36	33	39	0.201
Fasting glucose (mg/dL)	103 (92–138.5)	102 (90–133)	103.5 (92–146.5)	0.487
HbA1c (mmol/mol)	41 (37–51)	41 (36.3–46)	41 (37–57)	0.405
HOMA-IR	2.01 (1.19–3.88)	2.07 (1.27–4.03)	1.79 (1.11–3.77)	0.601
eGFR CKD-EPI (mL/min/1.73 m^2^)	82.4 ± 17.8	83.5 ± 17.1	81.3 ± 18.4	0.384
Triglycerides (mg/dL)	120 (81–167)	122 (80–167)	119 (81–169)	0.980
HDL-cholesterol (mg/dL)	55 (45.5–66.5)	52 (44–66)	56 (46–67)	0.329
LDL-cholesterol (mg/dL)	113.3 ± 40.0	115.0 ± 40.1	111.6 ± 39.3	0.560
CRP (mg/L)	1.9 (0.9–3.6)	2.3 (1.2–3.8)	1.4 (0.9–3.4)	0.031

Data are shown as means with standard deviation, median, and interquartile ranges, or as percentages, as appropriate. Comparisons between vitamin D and placebo groups were performed by a Student’s *t*-test, a Mann–Whitney U-test, or a chi-square test. BMI = body-mass index; B*P* = blood pressure; IGF-1 = insulin-like growth factor–1; 25(OH)D = 25-hydroxyvitamin D; PTH = parathyroid hormone; bAL*P* = bone-specific alkaline phosphatase; CTX = β-CrossLaps; P1N*P* = procollagen type 1 amino-terminal propeptide; HOMA-IR = homeostasis model assessment-insulin resistance; eGFR = estimated glomerular filtration rate; HDL-cholesterol = high-density lipoprotein-cholesterol; LDL-cholesterol = low-density lipoprotein-cholesterol; CR*P* = C-reactive protein.

**Table 2:** BTMs at baseline according to vitamin D level (i.e., <30, 30–39.8, 40–49.8, ≥50 nmol/L).

BTMs	25(OH)D < 30 (*n* = 4)	25(OH)D 30–39.8 (*n* = 31)	25(OH)D 40–49.8 (*n* = 22)	25(OH)D ≥ 50 (*n* = 111)	*p*-Value
25(OH)D (nmol/L)	9.9 (9.1–10.6)	13.4 (12.3–14.5)	18.3 (17.4–19.1)	24.8 (21.7–26.6)	<0.001
bALP (µg/L)	18.9 (15.1–21.4)	16.7 (12.6–22.5)	18.1 (14.7–22.7)	16.1 (13.8–19.7)	0.732
CTX (ng/mL)	0.13 (0.12–0.21)	0.13 (0.06–0.25)	0.17 (0.14–0.34)	0.19 (0.12–0.30)	0.126
OC (ng/mL)	10.8 (9.4–12.2)	11.5 (9.3–14.7)	14.6 (11.0–19.7)	12.9 (10.7–18.0)	0.218
P1NP (ng/mL)	31.5 (22.7–41.8)	36.2 (28.7–43.6)	43.6 (36.7–55.0)	41.2 (31.9–52.1)	0.103
PTH (pg/mL)	48.5 (26.7–88.0)	55.6 (41.7–68.9)	52.6 (37.9–64.3)	48.5 (41.0–61.9)	0.453

Data are shown as median and interquartile ranges; BTM = bone turnover marker; 25(OH)D = 25-hydroxyvitamin D; bAL*P* = bone-specific alkaline phosphatase; CTX = β-CrossLaps; OC = osteocalcion; P1N*P* = procollagen type 1 amino-terminal propeptide; PTH = parathyroid hormone.

**Table 3:** Correlations between 25(OH)D, 1,25(OH)2D3, PTH, and BTMs.

Vitamin D and BTMs	β and *n*	25(OH)D	1,25(OH)2D3	bALP	CTX	OC	P1NP	PTH
25(OH)D (nmol/L)	β	1.000						
	n	197						
1,25(OH)2D3 (pg/mL)	β	0.233 **	1.000					
	n	195	195					
bALP (µg/L)	β	−0.090	0.023	1.000				
	n	190	188	190				
CTX (ng/mL)	β	0.088	0.138	0.333 **	1.000			
	n	177	176	171	177			
OC (ng/mL)	β	0.084	0.066	0.419 **	0.626 **	1.000		
	n	195	193	189	175	195		
P1NP (ng/mL)	β	0.037	0.092	0.552 **	0.634 **	0.725 **	1.000	
	n	189	187	185	172	189	189	
PTH (pg/mL)	β	−0.086	0.108	0.115	−0.012	0.080	0.062	1.000
	n	197	195	190	177	195	189	197

Spearman correlations for 25(OH)D, 1,25(OH)2D3, PTH, and BTMs. BTM = bone turnover marker; β = correlation coefficient; *n* = number of patients with available data; 25(OH)D = 25-hydroxyvitamin D; bAL*P* = bone-specific alkaline phosphatase; CTX = β-CrossLaps; OC = osteocalcin; P1N*P* = procollagen type 1 amino-terminal propeptide; PTH = parathyroid hormone; ** = significant correlation.

**Table 4:** Intervention: BTMs at baseline and follow-up in study participants with available values at both study visits in all patients, in men and in women.

BTMs	Baseline	Follow-Up	Treatment Effect	*p*-Value
bALP (µg/L)				
Vitamin D (*n* = 94)	15.3 (12.3–18.7)	16.2 (12.7–19.6)	0.013 (−0.029 to 0.056)	0.533
Placebo (*n* = 96)	16.7 (13.3–20.6)	16.2 (13.5–21.1)		
CTX (ng/mL)				
Vitamin D (*n* = 85)	0.17 (0.09–0.27)	0.18 (0.11–0.28)	0.024 (−0.163 to 0.210)	0.802
Placebo (*n* = 86)	0.19 (0.11–0.29)	0.21 (0.10–0.33)		
Osteocalcin (ng/mL)				
Vitamin D (*n* = 85)	11.7 (9.0–16.6)	12.8 (9.5–17.7)	0.020 (−0.062 to 0.103)	0.626
Placebo (*n* = 86)	13.0 (9.1–18.0)	13.6 (9.7–20.2)		
P1NP (ng/mL)				
Vitamin D (*n* = 85)	34.9 (26.2–49.0)	38.6 (27.6–51.3)	−0.021 (−0.099 to 0.057)	0.597
Placebo (*n* = 86)	39.7 (31.4–51.0)	43.1 (32.3–56.3)		

Data are shown as median with interquartile ranges. Treatment effects with 95% confidence interval and *p*-values were calculated by ANCOVA for group differences at follow-up with adjustment for baseline values. BTM = bone turnover marker; 25(OH)D = 25-hydroxyvitamin D; bAL*P* = bone-specific alkaline phosphatase; CTX = β-CrossLaps; OC = osteocalcin; P1N*P* = procollagen type 1 amino-terminal propeptide.
